# Collagen V-induced nasal tolerance downregulates pulmonary collagen mRNA gene and TGF-beta expression in experimental systemic sclerosis

**DOI:** 10.1186/1465-9921-11-1

**Published:** 2010-01-04

**Authors:** Ana Paula P Velosa, Walcy R Teodoro, Daniel M dos Anjos, Renata Konno, Cristiane C Oliveira, Maria LH Katayama, Edwin R Parra, Vera L Capelozzi, Natalino H Yoshinari

**Affiliations:** 1 Rheumatology Division of the School of Medicine of the University of São Paulo (FMUSP), São Paulo, SP, Brazil; 2 Department of Radiology, Discipline of Oncology of the School of Medicine of the University of São Paulo (FMUSP), São Paulo, SP, Brazil; 3 Department of Pathology of the School of Medicine of the University of São Paulo (FMUSP), São Paulo, SP, Brazil

## Abstract

**Background:**

The purpose of this study was to evaluate collagen deposition, mRNA collagen synthesis and TGF-beta expression in the lung tissue in an experimental model of scleroderma after collagen V-induced nasal tolerance.

**Methods:**

Female New Zealand rabbits (N = 12) were immunized with 1 mg/ml of collagen V in Freund's adjuvant (IM). After 150 days, six immunized animals were tolerated by nasal administration of collagen V (25 μg/day) (IM-TOL) daily for 60 days. The collagen content was determined by morphometry, and mRNA expressions of types I, III and V collagen were determined by Real-time PCR. The TGF-beta expression was evaluated by immunostaining and quantified by point counting methods. To statistic analysis ANOVA with Bonferroni test were employed for multiple comparison when appropriate and the level of significance was determined to be *p *< 0.05.

**Results:**

IM-TOL, when compared to IM, showed significant reduction in total collagen content around the vessels (0.371 ± 0.118 vs. 0.874 ± 0.282, *p *< 0.001), bronchioles (0.294 ± 0.139 vs. 0.646 ± 0.172, *p *< 0.001) and in the septal interstitium (0.027 ± 0.014 vs. 0.067 ± 0.039, *p *= 0.026). The lung tissue of IM-TOL, when compared to IM, showed decreased immunostaining of types I, III and V collagen, reduced mRNA expression of types I (0.10 ± 0.07 vs. 1.0 ± 0.528, p = 0.002) and V (1.12 ± 0.42 vs. 4.74 ± 2.25, p = 0.009) collagen, in addition to decreased TGF-beta expression (p < 0.0001).

**Conclusions:**

Collagen V-induced nasal tolerance in the experimental model of SSc regulated the pulmonary remodeling process, inhibiting collagen deposition and collagen I and V mRNA synthesis. Additionally, it decreased TGF-beta expression, suggesting a promising therapeutic option for scleroderma treatment.

## Background

Progressive Systemic Sclerosis (SSc) is an autoimmune disease of unknown pathogenesis, characterized by the increased extracellular matrix (ECM) synthesis, vascular remodeling and autoantibody emergence, which results in scarring in multiple organs. The lung is usually involved, and is the main cause of mortality in this disease [1]. Interstitial lung fibrosis, of variable intensity, affects approximately 90% of patients, and the frequency of pulmonary hypertension varies from 5% to 35% [1]. A diagnosis of SSc has important prognostic implications owing to the clinical course marked by inexorable deterioration. Currently, no medical therapies have proved to prolong life expectancy. Thus, there is great interest in understanding lung involvement in SSc and the effects of treatment to avoid irreversible scarring and decreased survival. Although the exact mechanism of treatment effects remains unknown, the influence of immune inflammatory cells and their mediators is diminished in animal models [2,3], thus affecting collagen synthesis and degradation and interfering with ECM remodeling.

Because ECM remodeling is thought to promote pulmonary restoration, a group of collagens have been targeted as potentially useful indicators of ECM remodeling [4,5]. Specifically, collagen V is a promising indicator [6]. Collagen V is a highly conserved molecule among different animal species [4,5] and is normally found in lung ECM, composing the heterotypic fibrils with types I and III collagen. Collagen V is a minor collagen fraction not normally exposed in the tissues [7-10], retaining the amino- and carboxy-terminals, making it quite immunogenic.

Previously, we discovered an experimental model of SSc by immunizing healthy New Zealand rabbits with human collagen V emulsified with Freund's adjuvant. This resulted in intense inflammation of the lung and progressive ECM remodeling of the septal and bronchovascular axis [11]. The examination of other organs usually affected in SSc, such as skin, esophagus, kidney, heart and synovial membrane, showed identical and intense ECM remodeling [12-14]. In addition, several immunological alterations were observed, such as the presence of types I, III and IV anti-collagen antibodies, circulating immune complexes, and the emergence of antinuclear antibodies (ANA) and anti-Scl-70 antibodies [15]. Based on Sakkas's works [16,17] suggesting that SSc pathogenesis is related to the activation of T cells by still unidentified antigens, we postulated that collagen V usually found hidden between collagen I and III in heterotypic fibers, but exposed in our experimental model, could be one of the antigens responsible for triggering the T-dependent response (Th2). The activated Th2 cells and the IL-4 and IL-17 cytokines generated by their activation would explain the SSc triad: increased ECM synthesis, vascular remodeling and autoantibody production [17]. These alterations associated with the immunogenic role of collagen V make our experimental model important to test tolerance induction in the treatment of SSc. Considering that we have already demonstrated the efficacy of nasal tolerance with collagen V in skin remodeling of animals with SSc [18], in the present study we evaluated the amount of collagen deposition, mRNA collagen synthesis and TGF-beta expression in pulmonary septal and bronchovascular interstitium of rabbits after collagen V-induced nasal tolerance in experimental SSc. It was hypothesized that collagen V-induced nasal tolerance decreases the density of pulmonary perivascular and septal collagenous fibers.

## Methods

### Collagen V Immunization

Experimental SSc was induced in healthy New Zealand female rabbits (N = 12) with a mean weight of 2.50 Kg and 2 months of age. The complete immunization protocol includes 4 inoculations. The first is a subcutaneous (sc) injection with 1 mg of Col V isolated from human placenta [11-15], diluted in 1 ml of 10 mM acetic acid and added to an equal amount of complete Freund's adjuvant (Sigma Chemical Co.; St. Louis, Missouri, USA). The second inoculation occurs after 30 days and the animals received an identical subcutaneous injection. Fifteen days after the second subcutaneous injection, the rabbits received one reinforcement dose of 1 mg of Col V plus 1 ml incomplete Freund's adjuvant intramuscularly (third inoculation). Finally, a second identical reinforcement (fourth inoculation) is administrated after another 15 days [11-15]. The control group (N = 6) was inoculated with Freund's adjuvant diluted in 10 mM of acetic acid, following the same protocol of the immunized animals.

### Collagen V-Induced Nasal Tolerance

Nasal tolerance was induced in a group of six collagen V-immunized animals, through the nasal administration of daily doses of 25 μg of collagen V diluted in 25 μl of 10 mM acetic acid (**IM-TOL**). The nasal tolerance induction was initiated 150 days after the first immunization, and conducted for 60 days. Another group of six immunized animals (**IM**) was not tolerated. The control group (n = 6), inoculated with Freund's adjuvant (**CT-FA**) was tolerated by nasal route with collagen V, initiated 150 days after immunization. All animals were sacrificed at 210 days.

The animal procedures were approved by the Ethics Committee in Research, CAPPesq of the Clinical Board of the School of Medicine, University of São Paulo, as stated in Protocol of Research number 268/05.

### Collagenous Fibers Histomorphometric Analysis

To characterize the collagenous fibers in peribronchovascular and septal pulmonary interstitium, Masson's trichrome was used to stain the collagen-containing fibers in blue. Also, the Picrosirius staining method [19] observed under polarized light was used to intensify the normal birefringence of collagenous fibers and to determine the location of collagen-containing fibers. The number of collagen fibers in lungs was determined by an image analysis system in an optical microscope equipped with a light polarizer coupled to an image analyzer. The system consisted of a Q-Color 5 camera, coupled to an Olympus microscope, from which the images could be visualized on the monitor. The images were processed through a digital system installed in a computer (Pentium 4, 300 Mhz) using the Image-ProPlus, version 6.0 software. The enhancement of collagen birefringence promoted by the Picrosirius polarization method is specific for collagenous structures composed of aggregates of orientated molecules. The threshold for collagenous fibers was established for each slide after enhancing the contrast up to a point at which the fibers were easily identified as birefringent (collagen) bands. The area occupied by the fibers was determined by digital densitometric recognition, by adjusting the threshold level of measurement to all the fibers of the collagenous system. The collagen content was measured in the peribronchovascular and septal interstitium and expressed as a relationship between the quantities of collagen fibers divided by the total area of interstitium studied. The area of septal and bronchovascular interstitium in each specimen was carefully measured in the image analysis system using a cursor that allowed the free determination of the area between the basement membrane (septal interstitium) and the periadventitial layer (bronchovascular interstitium). The results express the amount of fibers of the collagenous systems (in area) per total area of interstitium, expressed as a fraction.

### Collagen I, III and V Immunofluorescence

Transversal sections of rabbit lungs prepared in slides that were previously treated with 3 - aminopropiltriethoxy Silano (Sigma Chemical Co., St. Louis, MO, USA) were immersed in hot (60°C) xylol for 20 min and then submitted to three cold xylol washings and hydrated with successive washings in ethanol, at decreasing concentrations (100%-75%), distilled water and phosphate buffer (PBS). For the exposition and recovery of the antigenic sites, the material was digested with pig pepsin (10,000 U/ml) (Sigma Chemical Co.) dissolved in 1 mM acetic acid, for 30 min at 37°C. The treated sections were washed three times, for 10 min each, with PBS and incubated with type I or V anti-collagen mouse polyclonal antibody, diluted at 1:50 in PBS, and type III anti-collagen monoclonal antibody (Calbiochem), with a 1:50 dilution during the night. After this incubation, the cuts were washed in PBS with 0.05% Tween_20 _and incubated for 90 min with anti-IgG mouse secondary antibody conjugated with fluorescein (Sigma Chemical Co.) diluted at 1:50 in a PBS solution, containing 0.006% Evans blue and mounted with a buffered glycerol solution. The reaction was visualized in a Nikon fluorescence microscope.

### Collagen I, III and V Real-time PCR (RT-PCR)

Selected specimens from peripheral areas of the lower pulmonary lobe were pulverized (Bio-Pulverizer™ BioSpec Products Inc., Oklahoma, USA) under liquid nitrogen and total RNA was isolated using Trizol reagent (Invitrogen Corporation, Carlsbad, CA, USA), according to the manufacturer's protocol. RNA quality and integrity were verified by the absorbance 260 nm:280 nm ratio (A_260/280_), which varied between 1.78 and 2.0, and through observation of 28S/18S rRNA on agarose gel (1%) electrophoresis, in denaturing conditions and visualization with ethidium bromide (ratio > 1.0).

Total RNA (4 μg) was reverse-transcribed using a hexamers primer (0.5 μg/μl) (GE Healthcare Life Sciences, Little Chalfont, St. Giles, UK) and Superscript III (Invitrogen Corp., Carlsbad, CA, USA). Real-time RT-PCR was conducted using SYBR-green I (Sigma Chemical Co.) in a Rotor-gene system (Corbett Research, Mortlake, Australia). Amplification reactions were conducted using 125 ng of cDNA, 1.25 U Platinum Taq Polymerase (Invitrogen), polymerase buffer (Invitrogen), 2.0 mM MgCl_2_, 200 μM each dNTP, 0.3 μM each primer, 5% DMSO and 0.1 μL SYBR^® ^Green. Amplification conditions consisted of denaturation at 95°C for 15 s followed by 40 cycles of annealing at 56°C for 60 s, and extension at 72°C for 60 s.

Primer sets were designed based on the coding region closer to the 3' end of the gene using Primer3 (Table 1). Sequences, present in different exons preferentially separated by long introns, were selected according to sequences deposited at http://www.ncbi.nlm.nih.gov/nucleotide. BLAST analysis http://www.ncbi.nlm.nih.gov/blast was conducted to avoid non-specific product formation. To minimize self- and cross-dimer hairpin formation, homodimer melting temperatures were verified using the program OligoTech version 1.00, Copyright 1995 (Oligos Etc. Inc. & Oligo Therapeutics Inc.).

**Table 1:** Sequence and description of the genes selected for the study

*Gene*	Genbank Accession Number	Primer sense (5'-3')	Primer antisense (5'-3')	Product size (pb)
COL I	AY633663	CTTGGGGTTCTTGCTGATGT	GGACCTCAAGATGTGCCACT	178
COL III	S83371	ATGTGTTTGGTGGAACAGCA	TGGCCCTGTTTGCTTTTTAT	204
COL V	AF451329	GTCCCCCTCAAACACTTCCT	TCTCAGCGTCCACAAGAAAA	154
GAPDH	AB231852	GTGAGTTTCCCGTTCAGCTC	AGGTCATCCACGACCACTTC	202

All samples were tested in duplicate and analyzed by the software Rotor-Gene 6 System (Corbett Research). Results displaying variation in CT - the cycle number at which logarithmic PCR plots cross a calculated threshold line - of less than 1.5 were used to calculate average values.

Data were expressed as CT values. Relative expression of genes of interest was normalized to that of GAPDH, and gene expression in each sample was then compared with expression in pool cells. The comparative CT method (ΔΔCT) was used for the quantification of gene expression, and relative expression was calculated as 2^-ΔΔCT ^[20].

### TGF-beta Expression

To evaluate TGF-beta in pulmonary septal and peribronchovascular interstitium, 4-μm paraffin sections were immunohistochemically stained with goat polyclonal TGF-beta (Santa Cruz Biotechnology Inc.; dilution 1:100) according to the labeled Streptavidin-Biotin Complex method used previously in others' works [21]. To quantify stained cells, a point-counting stereologic method [19] was employed using a reticulum formed by 100 points and 50 lines, each measuring 25 μm in length, adapted to a conventional microscope. At 400× magnification, the vessels and septal interstitium in each field were calculated according to the number of points hitting connective tissue, as a proportion of the total grid area. Then, we counted the number of positive cells within the pulmonary interstitium area. The TGF-beta expression was determined as the number of positive cells in each field divided by the interstitium area. The final results were then transformed to cells/mm^2 ^by adjusting the units.

### Statistical analysis

Differences between the groups were determined by the Shapiro-Wilks test to determine normality and Levene's one-way test for the homogeneity of variance. Independent-samples *t *test for two comparison and ANOVA with Bonferroni test for multiple comparison were performed when appropriate. All statistical procedures were performed with SPSS version 10.0 statistical software for Windows^® ^(Norusis M.J., SPSS, Inc., Chicago, IL). The level of significance was determined to be *p *< 0.05.

## Results

**Figure 1:**
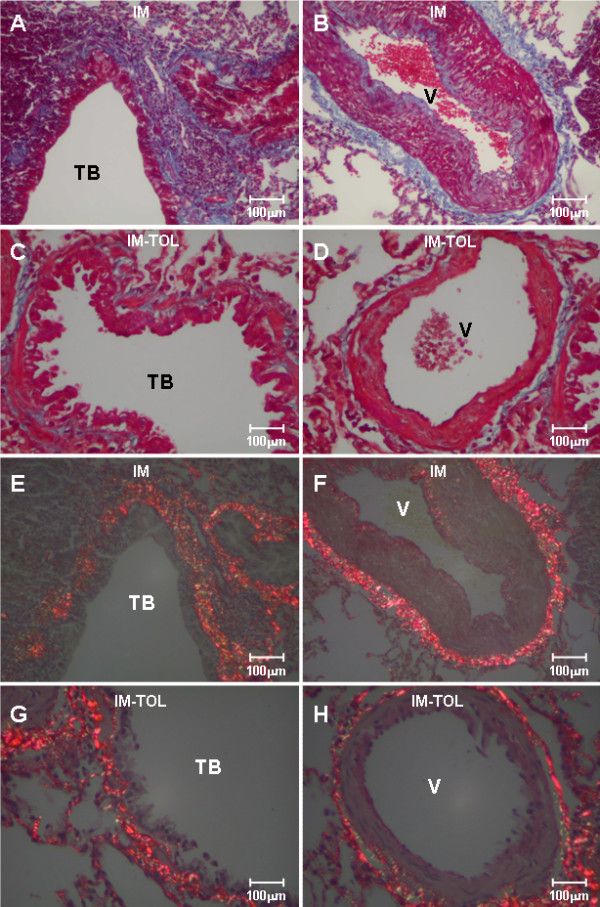
**Transversal sections of lungs of immunized rabbits, and lungs after nasal tolerance induction with collagen V, stained by Masson trichromic (A-D) and Picrosirius (E-H)**. In the immunized animals, thickening of the extracellular matrix is observed, with radial and periaxial distribution (A, B, E, F). After the induction of nasal tolerance with collagen V, there was a decrease in collagen deposition (G, H). TB = terminal bronchiole; V = vessel; Groups: IM = immunized; IM-TOL = immunized and tolerated; Magnification: 200×.

Figure 1 shows, respectively, lung samples obtained from immunized and tolerated animals stained by Masson's trichrome and Picrosirius under polarized light. Lungs of rabbits examined 210 days after the first inoculation (Figure 1A, B) presented prominent thickness of the septal and bronchovascular interstitium and increased reddish-yellow birefringence, indicating the presence of thick fibers, characteristic of the fibrotic process (Figure 1E, F). In contrast, lungs from tolerated animals show preservation of septal and peribronchovascular interstitium thickness (Figure 1C, D) coincident with the weak yellow birefringence of the fibers (Figure 1G, H).

**Figure 2:**
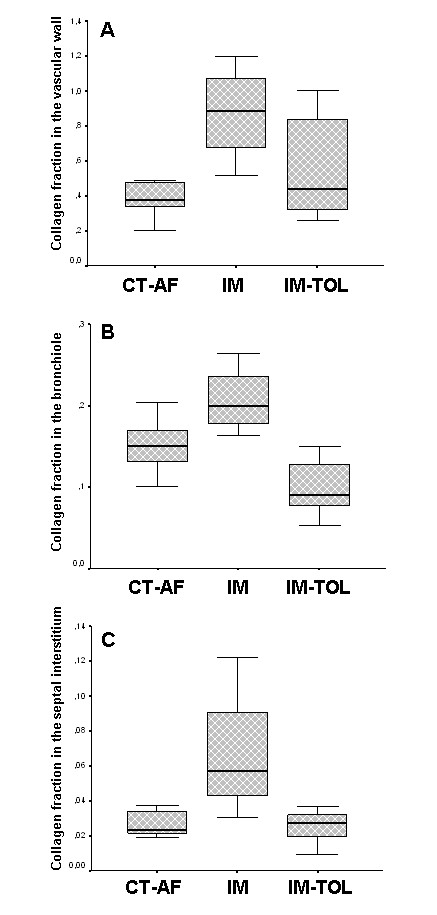
** Charts A, B and C show the content of collagen fibers among the groups**. A significant decrease in collagen fiber density in the vascular wall (p < 0.001) (A), in the bronchioles (p < 0.001) (B) and the pulmonary interstitium (p = 0.026) (C) was observed in tolerated animals, when compared to immunized ones. No difference was observed in the content of collagen between control and tolerated animals. Groups: CT-FA = Freund's adjuvant control; IM = immunized; IM-TOL = immunized and tolerated. Statistical analysis was employed by ANOVA with Bonferroni test.

The density of the collagen fibers is decreased around the vessels (0.371 ± 0.118 vs. 0.874 ± 0.282; *p *< 0.001), bronchioles (0.294 ± 0.139 vs. 0.646 ± 0.172; *p *< 0.001) and in the septal interstitium (0.027 ± 0.014 vs. 0.067 ± 0.039, *p *= 0.026) in tolerated animals when compared to the immunized ones (Figure 2A, B and 2C).

**Figure 3:**
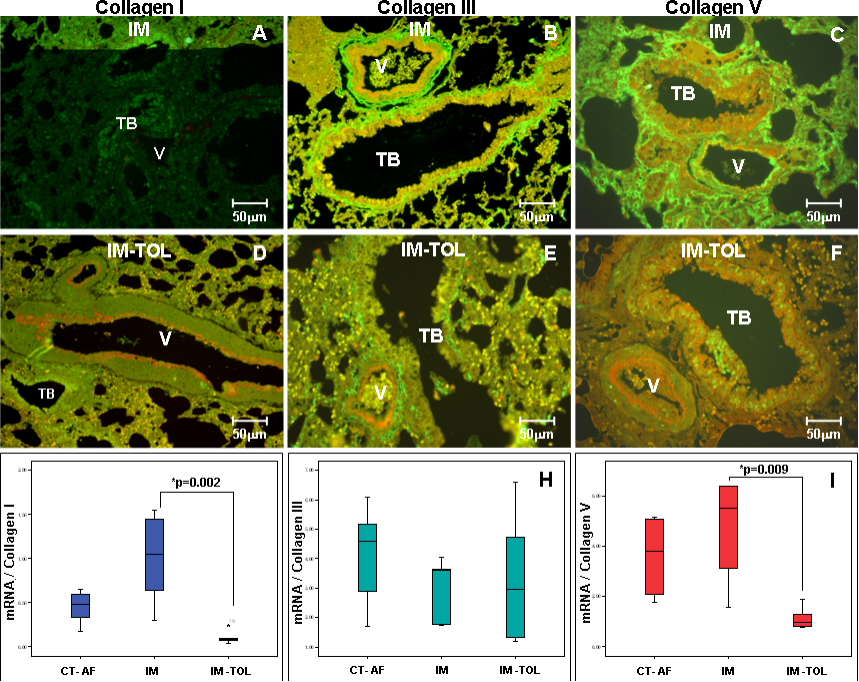
** Panels A to F show the lungs of rabbits immunolabeled with types I, III and V collagen by immunofluorescence**. A decrease in the expression of collagens I (D), III (E) and V (F) in the peribronchovascular interstitium and in the septal interstitium of tolerated animals was observed when compared to immunized animals (A, B and C, respectively). Panels G, H and I show the differential gene expression of the mRNA for collagens I, III and V, respectively, in lung tissue of control and immunized animals and after the induction of nasal tolerance with collagen V. The box plot shows the distribution of all values between the bars (quartiles 25, 50 and 75 within the box), except extreme values (°1.5- to 3.0-fold the dimension of the box of the 75th percentile; *values that are more than 3-fold the dimension of the box of the 75th percentile). The Bonferroni test was used, and we considered the gene to be differentially expressed where p ≤ 0.05. TB = terminal bronchiole; V = Vessel; Magnification: A-F, 400×. Groups: CT-FA = Freund's adjuvant control; IM = immunized; IM-TOL = immunized.*Statistical significance.

The immunolabeling for collagen I in the lung tissue of immunized animals showed a dense and heterogeneous pattern of fluorescence, more intense around the peribronchovascular interstitium than along the septal interstitium (Figure 3A). The immunoexpression of collagen III was equally intense along the bronchovascular interstitium of immunized animals, mainly in the adventitia of the pulmonary artery (Figure 3B). As for the expression of the collagen V, the immunized animals showed intense labeling, seen as thick fibers along the bronchovascular interstitium and the septal interstitium, thus differing from its normal fibrillar pattern of thin fibers (Figure 3C). In the group tolerated with collagen V, the lung tissue presented a homogeneous labeling pattern, characterized by the decreased fluorescence intensity for collagen I in the bronchovascular interstitium and the septal interstitium (Figure 3D), as well as decreased expression of collagen III, with a thin fiber pattern in all analyzed regions (Figure 3E). The expression of collagen V in the group of tolerated animals shows a reversion to the thin fibrillar pattern, characteristic of the expression of this type of collagen in the bronchi and vessels of these animals (Figure 3F).

The expression of mRNA in lung tissue is significantly decreased in the group of animals tolerated with collagen V (IM-TOL), when compared to the animals that were only immunized (IM), for types I (0.10 ± 0.07 vs. 1.0 ± 0.528, p = 0.002) and V (1.12 ± 0.42 vs. 4.74 ± 2.25, p = 0.009) collagen. There is no significant difference in the expression of collagen I between tolerated (IM-TOL) and control (CT-FA) groups (p = 0.357). A marginal significance was found for lower mRNA collagen V expression in tolerated (IM-TOL) group compared to control (CT-FA) (p = 0.073). Collagen III mRNA expression showed no difference between tolerated (IM-TOL) and immunized (IM) animals (3.2 ± 2.17 vs. 3.07 ± 1.03, p = 0.628) (Figure 3G, H and 3I).

**Figure 4:**
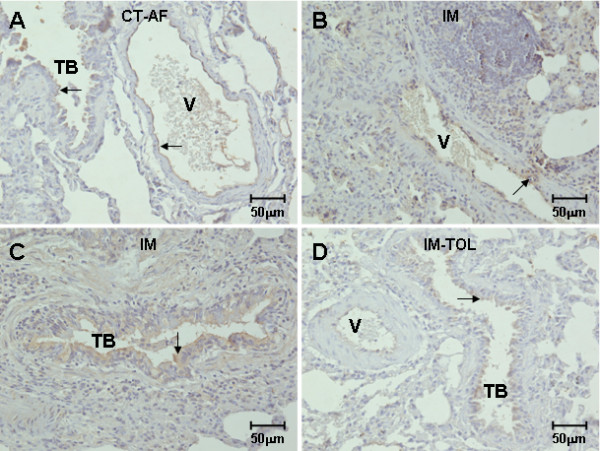
** Rabbit lungs immunolabeled with TGF-beta by immunoperoxidase**. A decrease in the expression of TGF-beta in the vascular endothelium cells and bronchial epithelium of tolerated animals (D) was observed when compared to immunized ones (B, C). There was no significant difference in immunolabeling for TGF-beta between tolerated (D) and control animals (A). TB = terminal bronchiole; V = vessel; Magnification: 400×. Groups: CT-FA = Freund's adjuvant control; IM = immunized; IM-TOL = immunized and tolerated.

Figure 4A shows the slight expression of TGF-beta in the endothelium and epithelium of control lungs contrasting with the significant cytokine labeling in the endothelium and epithelium in immunized animals (Figure 4B, C; Table 2). In tolerated animals, one can observe a significant attenuation of the TGF-beta cytokine expression in the endothelium and epithelium (Figure 4D, Table 2).

**Table 2:** The TGF-beta expression in vessels and septal interstitium from lungs of control, immunized and type V-induced nasal tolerance rabbits groups

	CONTROL	IMMUNIZED	TOLERATED	P
**VESSEL**				
*Endothelium cells*	6.7 ± 3.85	43.5 ± 5.7	10.77 ± 4.3	*p < 0.0001
*Smooth muscle cells*	11.04 ± 1.0	53.68 ± 4.06	9.93 ± 3.77	*p < 0.0001
**SEPTAL INTERSTITIUM**				
*Epithelial cells*	5.04 ± 2.46	13.65 ± 1.39	6.03 ± 1.47	*p < 0.0001
*Interstitial Fibroblasts*	8.55 ± 1.96	20.13 ± 1.60	11.55 ± 1.88	*p < 0.0001

* p = immunized vs. tolerated; ANOVA with Bonferroni test was employed to statistical analysis.

The results are reported as cells/mm^2^.

## Discussion

Currently, no medical therapies can alter the disease course of SSc, especially in the presence of pulmonary complications. SSc is not cured with immunosuppressive therapies, and may have its clinical course marked by serious adverse effects, such as medullary depression, systemic infections, hepatopathy, or nephropathy. Drugs employed in the treatment of pulmonary hypertension, such as endothelin inhibitors, are expensive and have restricted use, and, thus, are not routinely prescribed.

In the present study, we demonstrated that the treatment of pulmonary complications in animals with SSc through the induction of nasal tolerance with collagen V, showed similar results to those previously obtained in rabbit skin, when submitted to this procedure [18]. We didn't find a progression of the disease process which was characterized by a decrease in interstitial fibrosis and vascular sclerosis, as well as lower expression of collagens I and V and pro-fibrotic cytokines. Tolerance induction inhibits the systemic immunological response through the administration of specific antigens, which are important in the pathogenesis of immunological diseases, via the mucosa (nasal or oral), leading to the prevention and/or treatment of autoimmune and allergic diseases or preventing transplant rejection [22].

The mechanisms of tolerance induction depend on the dose used. High doses induce T-cell deletion or anergy, and low doses promote regulatory T-cell activation (regT), such as TH3, which produces TGF-beta and Tr1 cells that generate cytokines IL-10 and IL-4; both of these cytokines have immunosuppressive activity [23-27]. Thus, for all these reasons, we should not to be surprised to learn that collagen V nasal tolerance didn't allow the progression of the fibrotic process and the pulmonary and vascular remodeling, and our results now confirm the therapeutic importance of collagen V role in an experimental model of SSc.

We also confirmed the improvement in the histological parameters by molecular analysis. In fact, animals submitted to nasal tolerance induction with collagen V showed a decrease in the mRNA expression for collagens I and V. We believe that the collagen III expression showed no difference between only immunized and tolerated animals during the fibrosis period, since this collagen is normally expressed in more initial fibrosis. The collagen synthesis normalization in the experimental animals after the tolerance induction with collagen V, discloses, for the first time, the possibility of avoid fibrosis progression in an SSc experimental model. The present study also shows that the fibrosis observed in the experimental model of SSc may be structurally different from the physiological fibrosis of tissue repair. In the physiological healing processes, which are considered irreversible, the proportions of collagens I, III and V do not change. This differs from the remodeling process in SSc, where there is a disproportionate expression of collagen V. Further studies in randomized and prospective trials will be necessary to determine whether this defective tissue architecture exhibited by animals with SSc is the consequence of an aberrant fibroblast or if there is an atypical molecular arrangement of the collagens that constitute the heterotypic fibers (collagens I, III and V), resulting from the deviant expression of collagen V.

This report provides previously undescribed morphological insight into the pathogenesis of SSc and expands the scope of diseases associated with autoantigens, such as collagen V. We predict that collagen V is one of the antigens involved in the activation of Th2 subtype lymphocytes, which initiates the synthesis of pro-fibrotic cytokines and stimulates the immunological system to produce autoantibodies [16,17]. The nasal tolerance mechanism with collagen V inhibits this anomalous immunological response, leading to the normalization of the inflammatory process and thus avoiding the anomalous matrix remodeling. We also demonstrated the excessive production of TGF-beta, the main pro-fibrotic cytokine, and its normalization after the nasal tolerance induction with collagen V. In an autoimmunity model such as SSc presenting a severe fibrotic involvement of organs, the decrease in TGF-beta is interesting, since this cytokine is fibrogenic [28].

The above considerations have support in the literature. Recently, several authors confirmed that collagen V is, in fact, an autoantigen, also capable of inducing lung transplant rejection in a murine experimental model [7,8,29-33]. Yoshida *et al *[32] also showed the importance of specific T cells sensitized for collagen V in alloimmunity and auto-immunity in a murine model of lung transplant. They demonstrated that the oral tolerance with collagen V was capable of inhibiting the acute rejection of the lung graft, preventing the development of bronchiolitis obliterans, a main causal factor of death in lung transplants in mouse experimental models as well as in transplanted humans [32-34]. In addition, Mizobuchi *et al *[35] identified regulatory T cells (CD4^+^CD45RC^higth^) that mediated tolerance for collagen V in lung transplants in an experimental rat model.

To date, the therapeutic approach by inducing immune tolerance obtained in our model cannot be directly transferred into the human situation without any precautions. However, few studies published in the literature show that patients that have been tolerated with bovine collagen I by the oral route exhibit a decrease in the T-dependent immune response for in vitro collagen I and a considerable clinical improvement [36]. More recently, Postlethwaite et al [37] demonstrated that oral tolerance induction with type I bovine collagen, administrated for a period of 15 months, significantly decreased the skin thickening in patients with more advanced manifestations of SSc. More studies are necessary to suggest that tolerance with collagen V could be an alternative approach for the treatment of human SSc such as collagen I.

## Conclusions

We conclude that collagen V-induced nasal tolerance is an effective therapeutic procedure in reducing inflammation and remodeling that occurred at the cost of collagen deposition in the lungs of animals with SSc. The fact that the progression of the fibrotic disease can be avoided in the SSc experimental model predicts remarkable advances in the treatment of this severe disease.

## Abbreviations

**SSc: **Systemic sclerosis; **ECM: **Extracellular matrix; **mRNA: **messenger; **TGF-**beta: Transforming growth factor; **IM: **Immunized; **IM-TOL: **Immunized and Tolerated; **CT-FA:** Freund's adjuvant control; **PCR: **Polymerase Chain Reaction; **RT-PCR: **Reverse Transcriptase Polymerase Chain Reaction; **ANA: **Antinuclear antibodies; **IL: **Interleukin; **PBS: **Phosphate buffered saline; **dNTP: **Nucleotides; **DMSO: **Dimethyl sulfoxide; **BLAST: **Basic Local Alignment Search Tool; **CT: **Cycle threshold; **GAPDH: **Glyceraldehyde-3-phosphate dehydrogenase; **ANOVA: **Analysis of variance between groups; **SPSS: **Statistical Package for the Social Sciences.

## Competing interests

The authors declare that they have no competing interests.

## Authors' contributions

APPV carried out the induction of the experimental scleroderma model and nasal tolerance and the immunofluorescence experiments, and drafted the manuscript; WRT contributed to conception and design of the original study and analysis and interpretation of histological, immunohistochemical and molecular data; DMA and ERP contributed to the acquisition of histologic and morphometric data and performed the statistical analysis; RK, CCO and MHK contributed to the acquisition of molecular data and performed the statistical analysis; VLC and NHY were involved in drafting the manuscript and revising it for important intellectual content. All authors read and approved the final manuscript.
